# The Effect of Vitamin Supplementation on Subclinical Atherosclerosis in Patients without Manifest Cardiovascular Diseases: Never-ending Hope or Underestimated Effect?

**DOI:** 10.3390/molecules25071717

**Published:** 2020-04-09

**Authors:** Ovidiu Mitu, Ioana Alexandra Cirneala, Andrada Ioana Lupsan, Mircea Iurciuc, Ivona Mitu, Daniela Cristina Dimitriu, Alexandru Dan Costache, Antoniu Octavian Petris, Irina Iuliana Costache

**Affiliations:** 1 Department of Cardiology, Clinical Emergency Hospital “Sf. Spiridon”, 700111 Iasi, Romania; 2 1st Medical Department, University of Medicine and Pharmacy “Grigore T. Popa”, 700115 Iasi, Romania; 3 Department of Cardiology, University of Medicine, Pharmacy, Science and Technology, 540139 Targu Mures, Romania; 4 Department of Cardiology, University of Medicine and Pharmacy “Victor Babes”, 300041 Timisoara, Romania; 5 2nd Morpho-Functional Department, University of Medicine and Pharmacy “Grigore T. Popa”, 700115 Iasi, Romania

**Keywords:** subclinical atherosclerosis, cardiovascular diseases, vitamins, antioxidants, inflammation, primary prevention

## Abstract

Micronutrients, especially vitamins, play an important role in the evolution of cardiovascular diseases (CVD). It has been speculated that additional intake of vitamins may reduce the CVD burden by acting on the inflammatory and oxidative response starting from early stages of atherosclerosis, when the vascular impairment might still be reversible or, at least, slowed down. The current review assesses the role of major vitamins on subclinical atherosclerosis process and the potential clinical implications in patients without CVD. We have comprehensively examined the literature data for the major vitamins: A, B group, C, D, and E, respectively. Most data are based on vitamin E, D and C supplementation, while vitamins A and B have been scarcely examined for the subclinical atherosclerosis action. Though the fundamental premise was optimistic, the up-to-date trials with vitamin supplementation revealed divergent results on subclinical atherosclerosis improvement, both in healthy subjects and patients with CVD, while the long-term effect seems minimal. Thus, there are no conclusive data on the prevention and progression of atherosclerosis based on vitamin supplementation. However, given their enormous potential, future trials are certainly needed for a more tailored CVD prevention focusing on early stages as subclinical atherosclerosis.

## 1. Introduction

Multiple observational studies have shown an inverse relationship between increased levels of micronutrients and cardiovascular diseases (CVD). However, most interventional and prospective trials have not clearly confirmed the link between them, thus, CVD prevention guidelines do not yet recommend an additional intake of micronutrients. In contrast, new current research targets the link between micronutrients and early stages of atherosclerosis, respectively their influence on inflammatory markers given their antioxidant capacity, aiming the subclinical atherosclerosis as the point where the produced damage may be still reversible or at least slowed down with favorable results over the targeted organs and systems.

Atherosclerosis, the leading cause of CVD, is an inflammatory pathology that begins when cholesterol-containing low-density lipoproteins (LDLc) accumulate in the vascular intima and activate the endothelium. A complex immunological mechanism leads to the secretion of pro-inflammatory cytokines that contribute to the local inflammatory process and increase the atherosclerotic plaque. It is a process with a long asymptomatic evolution; the moment when symptoms occur denotes the presence of advanced CVD. During the asymptomatic period of atherosclerotic progression, the subclinical organ damage can occur. By applying measures of primary CVD prevention during the subclinical atherosclerotic period, the incidence of CVD events can be decreased and slowed down, thus preventing the progression of pathological events [1].

Recent studies have focused on multiple mechanisms that appear to play key roles in the atherosclerotic inflammatory process, such as the oxidized LDLc that induces the recruitment of monocyte-macrophages to the endothelial wall, with highly atherogenic characteristics, the proliferation of smooth muscle cells induced by hyperglycemia, the oxidative stress and many others. Knowing the set off of these processes represents the finale purpose of these findings with the ultimate goal of stopping or slowing them down in order to cure or delay the atherosclerotic process [2]. Certain diets may lead to the development of metabolic syndrome with a direct effect on the evolution of inflammatory responses of the vascular intima. Other studies hinted that micronutrient-full diets directly influence the oxidative balance, the cellular growth and immune regulation [3]. Therefore, it appears that micronutrients, especially vitamins with antioxidant and anti-inflammatory properties, may play a key role in targeting the subclinical atherosclerosis by equilibrating the oxidation-antioxidation balance and metabolism [4]. However, though the premises were consistent in many observational studies, most antioxidant and vitamin supplementation trials have failed to show an incremental CVD effect. Nonetheless, few studies have assessed the potential vitamin role in primary prevention and, especially, starting from subclinical modifications as early marker of CVD.

In the current literature review, we aimed to assess the influence of major vitamins on the progression of subclinical atherosclerosis in patients without manifest CVD and their potential effect on reducing the CVD burden starting from subclinical disease. For this purpose, we have introduced certain keywords (*subclinical atherosclerosis, each vitamin separately, cardiovascular diseases, prevention*) in the major databases (PubMed, ScienceDirect, Web of Science). The obtained papers were further reviewed in order to exclude subject groups with manifest or atherosclerotic CVD while the remaining articles have been extensively analyzed.

## 2. Vitamins and Subclinical Atherosclerosis

### 2.1. Vitamin E

Vitamin E is an essential micronutrient that includes both tocopherols (TP) and tocotrienols (T3), which comprise antioxidants that are meant to modulate lipid peroxidation. It is found in plants, seeds and their derivatives. The most active isomer of this element is α-tocopherol, a very liposoluble phenol, with an important antioxidant action [5], making it particularly important in the human metabolism as it appears to be found in fat deposits, lipoproteins, and tissues with lipid-rich elements [6].

This vitamin appears to be involved in several stages of inflammation and immune regulation, modulating cell functions and gene expression [7]. It has protective effects against CVD, metabolic disorders and other diseases where oxidative stress represents a risk factor, by cancelling the oxidation produced by free radicals on cells, nervous tissues or membranes [8]. Due to the presence of tocotrienols, it has the effect of lowering cholesterol beyond the antioxidant mechanism [7,8,9].

Chronic and continuous inflammation is involved in the evolution of atherosclerosis. The oxidative stress produced inside the cell is a key mechanism in the oxidative damage of biomolecules. Thus, it is understandable why a clear link between vitamin E and subclinical atherosclerosis is under research, being widely suggested that it can provide a useful prognosis in CVD and metabolic disorders as well as a therapeutic agent in various pathologies. Many animal studies showed the beneficial effects of TP and T3 on vascular lesions of atherosclerosis, but in clinical trials the results were variable, most of them negative [10,11]. Though the reasons for these results were unclear, it was supposed that research animals can be better controlled and more rigorously measured than humans in trials [10]. The advanced stage of atherosclerosis or CVD was another hypothesis for the high doses of vitamin E that were required for showing an improvement in the studied pathology [11,12].

In the prevention field, though, vitamin E showed a better response. In the early stage of atherosclerosis, the required quantity was lower and, even though the patients could not be drastically controlled, the correlation between serum vitamin E and the decrease of LDLc and total cholesterol were easily observed [13]. Serum lipid concentration is a paramount factor in the development of atherosclerosis, dyslipidemia being strongly related to CVD.

Vitamin E, due to its antioxidant properties, protects hepatic cells against oxidative stress [14]. A recent study aimed to correlate circulating α- and γ-TP levels to adiposity-related characteristics by using high performance liquid chromatography and magnetic resonance imaging to quantify the liver fat and adipose tissue. Though the serum vitamin E level depends on the variable absorption in the gastro-intestinal tract [15], the vitamin status can be influenced by the liver metabolization and the oxidative stress. There was a significant association between the metabolic alterations and the serum concentration of the studied micronutrient [16,17].

Another study assessed the vitamin E supplementation for a 3-year period in two groups – smoking men, otherwise healthy, and post-menopausal women. The preventive effect was more obvious in the first group. This fact may be generalized to all male subjects, however, the lack of effect on women is not definitely, further studies being necessary in order to consider either this result may be due to statistical error or because this group has better protection against atherosclerotic development [18].

Trials revealed that patients with subclinical atherosclerosis (with risk factors but no CVD) that were treated with vitamin E showed significantly improvement of peripheral-artery disease and a decrease incidence of angina pectoris. No trial showed beneficial results on the carotid intima-media thickness (cIMT) progression in secondary prevention CVD patients [19].

Vitamin E has been one of the most studied antioxidants that may be involved in CVD prevention. Many observational and cohort studies suggested an inverse association between high dietary intake of vitamin E supplementation and CV events [20,21,22]. Though the postulated benefit was rather small to medium, there was a promising premise for implying vitamin E into CVD prevention. The antiatherogenic properties of vitamin E were suggested in its ability to decrease LDLc, diminish oxidative stress, reduce inflammation and inhibit vascular smooth muscle cell proliferation and expression of adhesion molecules [21].

Nonetheless, the interventional CVD trials of vitamin E supplementation presented divergent and modest positive results and did not fulfill the initial expectations [20,22]. As well, the association between CVD and serum or plasma TP level was rather inconsistent. Several hypotheses have been postulated for the lack of efficacity in interventional trials [22,23]. First, self-reported nutritional questionnaires are highly subjective and usually people tend to over-report their intake of fruits and vegetables or under-report other sources of vitamin E such as oil consumption. Secondly, it is difficult to quantify precisely the beneficial amount of vitamin E as there is a high bioavailability of TP and T3 in a mixture of foods that contribute to the daily nutrient intake and the interactions with other food ingredients could represent another issue. Further on, in nutritional interventional trials a great number of different CVD-associated parameters may appear and overrule the potential beneficial effect of vitamin E supplementation on CVD outcomes. On the other hand, high doses of vitamin E may exhibit prooxidant effect by enabling production of α-tocopheroxyl radical [21].

Most interventional trials failed when using vitamin E in secondary prevention but in primary prevention the effects seem to be encouraging. Thus, some interventional studies intended to assess the possible role of vitamin E supplementation on subclinical atherosclerosis as early marker of dysfunction as primary prevention CVD surrogate [18,24,25,26]. Though subclinical CVD modifications cannot be extrapolated to CVD outcomes, the results are encouraging and have been summarized in Table 1.

### 2.2. Vitamin D

Vitamin D is a group of lipophilic prohormones synthesized in the skin with the aid of ultraviolet-B radiation exposure and activated into the liver and kidneys by sequential hydroxylation or obtained by dietary means and absorbed by the lymphatic system from the proximal small intestine. It facilitates the absorption of calcium in the bowels and the deficiency of this vitamin can be associated with significant pathological changes in CV structure, mediating the link between parathyroid, kidneys and bones. Vitamin D therapeutic roles include improvements in endothelial function via anti-inflammatory actions [27].

Low vitamin D is related to a high CV risk, affecting proatherogenic serum cholesterol loading capacity, adipokine profile and subclinical atherosclerosis. Such results are achieved since these vasculotropic hormones present a wide expression of receptors in many body cells and organs [28].

The anti-atherosclerotic effect of vitamin D is explained by the positive impact on circulating lipoproteins functions, which play a key role in atherogenesis, rather than their serum concentration. The modulating effect on serum lipoprotein functions is closely linked to macrophage cholesterol homeostasis. Vitamin D also reduces the serum cytokines and adipokines, well known proatherogenic mediators, having a beneficial effect on adipokine profile, lipoprotein functions and vascular parameters [28].

By these mechanisms, vitamin D ameliorates vascular health and lowers the CV risk. Different studies have revealed that this hormone protects against atheromatous disease in patients with chronic kidney disease. In dialyzed patients, a connection between body mass index (BMI) and vitamin D was observed: lower odds of atheromatous progression were found in patients with low to moderate BMI but with higher levels of serum vitamin D [28].

Low serum concentration of 25-hydroxyvitamin D [25(OH)D] increased atherosclerotic and CV risk, being associated with dyslipidemia, high levels of serum LDLc and low levels of high-density lipoproteins (HDLc) in pre-menopausal healthy women [29,30]. The supplementation ameliorated serum lipoprotein functions, adipokine profile and subclinical atherosclerosis. As markers of atherosclerosis, standard measurement techniques were used, such as flow-mediated dilatation (FMD), pulse wave velocity (PWV) and augmentation index (AIx). The deficiency was defined as serum levels of 25(OH)D under 20 ng/mL. After the exclusion of patients with history of CVD and risk factors, or patients that received drugs that affect the endothelial functions such as statins, nitrates or angiotensin-converting enzyme inhibitors, the study concluded that vitamin D supplementation has a key role in lipoprotein functions in real life situation [31].

There are gender-differences in the action of vitamin D. A South Korean study that studied the serum levels of 25(OH)D on 404 men and 650 women revealed consistent discrepancies on metabolic components such as blood pressure, lipid profiles or glycemic index as well as on arterial changes such as brachial-ankle PWV (baPWV) or cIMT. While men did not present a particular correlation, women showed a connection between dietary vitamin D and triacylglycerol levels while both sexes showed a direct relationship between vitamin D and HDLc, but this study did not reveal a beneficial improvement on subclinical atherosclerosis despite vitamin D direct actions in the cholesterol metabolism [32].

Few studies covered the process of atherosclerosis in young people, given the idea that atherogenesis begins early in childhood. Risk factors, such as obesity, accelerate the atherosclerotic process, cIMT being a subclinical indicator of atherosclerosis, especially among overweight individuals [33]. Vitamin D concentration is associated with parathyroid hormone (PTH) that controls bone metabolism and has also receptors on arteries. Thus, high serum concentrations lead to hypertension, cardiac hypertrophy and endothelial dysfunction [34]. Moreover, vitamin D has effects on vascular smooth cell muscle, increasing the risk of atherosclerosis, while low concentration can lead to exacerbation of other CVD risk factors, such as arterial hypertension, obesity or diabetes. Even though there is limited evidence, a link between low 25(OH)D concentrations in childhood and increased cIMT in adulthood has been described. The study concluded that there may be a direct correlation between vitamin D insufficiency and subclinical atherosclerosis in overweight adolescents, but further investigations are needed in order to elucidate the possibility that PTH may contribute independently of 25(OH)D in the atherosclerotic process [34].

For quantifying the evidence that indicates the association between vitamin D and increased risk of CVD, the incidence of hospitalization for heart failure in patients with low concentration of 25(OH)D has been studied. Compared to normal levels of vitamin D, low serum concentration of 25(OH)D was associated with higher risk of heart failure hospital admission and with more days of hospitalization. It can be speculated that vitamin D cannot undo the existing lesions but may prevent further CVD evolution and decrease symptoms [35].

Though all these mechanisms, the supplementary intake of vitamin D was assumed to have a protective effect on CVD development. A large meta-analysis comprising more than 26.000 individuals from eight prospective cohort studies revealed a consistent association between 25(OH)D level and CV mortality in subjects both with and without a CVD history at baseline [36]. However, in primary CVD prevention, the vitamin D implications seem to diminish the clinical relevance. As a vitamin that is highly available, many studies tried to assess CVD outcomes or subclinical CVD modifications with an increase intake of vitamin D. The inconsistences of vitamin D trials could be attributable to heterogeneity in dosage or baseline concentrations, different non-accountable CVD biases or other concomitant treatment, study duration or design, or population type [21,37]. There is no standard consensus for vitamin D administration, especially for the CVD prevention.

The up-to-date study results of vitamin D supplementation on subclinical atherosclerosis are rather controversial (Table 2). There is no certain proof that vitamin D can induce regression of already pre-existing lesions and the effects of supplementation on different pathologies are not clarified but it has been demonstrated that it may ameliorate vascular health in subjects with low CV risk. Nonetheless, the genetic status and lifestyle factors may overrule the contribution of vitamin D regarding the risk of developing subclinical atherosclerotic disease [38].

### 2.3. Vitamin C

Vitamin C, also known as ascorbic acid, in an essential water-soluble nutrient with antioxidant properties. The mechanism by which cellular DNA, lipids and proteins are protected from the oxidative stress and the damage of radical oxygen species (ROS) is still unknown. It is certain that the anti-inflammatory properties of vitamin C explain the preventive effects of the diets rich in this nutrient in CVD or cancer [50]. Ascorbic acid has a key role in many physiological processes such as antioxidation, antiinflammation and oxidative stress. The vitamin C property of donating electrons is closely linked to all of its known functions [50].

Low plasma concentrations of vitamin C inevitably lead to scurvy. Signs and symptoms of this disease are related to the well-known enzymatic actions of ascorbate, such as the difficulty of collagen binding that results in loss of teeth and tissue damage, as a reflection of connective tissue destruction [51]. This is due to the ascorbic acid properties that participate in the synthesis of cholesterol, peptide hormones, amino acids, catecholamines and carnitine. Though these enzyme actions have not been unequivocally demonstrated in vivo, there must be some biochemical pathways that specifically require vitamin C, otherwise scurvy could be cured without ascorbate [52].

The enzymes used in the hydroxylation of lysine and proline (residues of collagen) – reaction which is a mandatory process for the evolving of the collagen triple helix physiological evolution – are closely linked with ascorbic acid [53,54].

Many diseases including atherosclerotic CVD or cancer may appear as a cause of the oxidative damage of the cells [50,55]. The genesis of atherosclerosis depends on multiple factors that are present alongside with other CV comorbidities such as diabetes mellitus, metabolic syndrome, dyslipidemia and arterial hypertension [56,57].

Different studies revealed that the use of antioxidant vitamin supplementation might reduce the CVD risk. Initial results showed the positive connection between the vegetable and fruit intake and improvement in CV outcomes. Further data demonstrated that the patients consuming such food had a lower risk of coronary heart disease (CHD) unlike people who were on the lowest quintile of fruits and vegetables intake [58]. The next step was pointing out which nutrient has the highest effect on CV physiology. Vitamin C was thoroughly studied because of its antioxidant capabilities and the direct effect on oxidized LDLc, which is quickly incorporated into the atherosclerotic plaque by scavenger receptors [59,60]. These findings made the LDLc oxidation as target of prevention, with vitamin C showing a potential role in CVD prevention. As well, it presents the capacity of reducing the monocytes endothelial adhesion, mechanism that is considered to be one of the early signs in the development of atherosclerosis. This hypothesis has been explored in cigarette smoking individuals, revealing that restoring the plasma vitamin C concentration in people that smoked one to two packs of cigarettes per day decreased the ability of monocytes to adhere on the endothelial wall as compared to the values found in non-smokers [61].

Additionally, ascorbic acid showed an increased action on nitric oxide, improving the latter production and reducing blood pressure. A study conducted on younger and older healthy adults demonstrated a significant reduction of arterial stiffness by measuring the PWV but there was a controversial lowering of systolic and diastolic blood pressure in older subjects and an increase of diastolic blood pressure in the younger patients when compared a regime of acute vitamin C administration to a chronic supplementation. The acute vitamin C administration was composed of a bolus of 0.06 g/kg fat-free mass (FFM) dissolved in saline and infused for 20 min, followed by a “drip infusion” of 0.02 g/kg FFM over 60 min while the oral supplementation was administrated for 30 consecutive days, 500 mg/day. Another novel aspect was represented by the spectacular increase of ascorbic acid concentration in younger subjects with initial lower concentrations as compared to the older subjects, but a clear explanation has not been defined yet. A plausible hypothesis might be that the renal function is reduced with age, resulting in slower kidney excretion of the nutrient excess in older subjects. However, further investigation on this matter is required [62].

Moreover, vitamin C appears to prevent the apoptosis of vascular smooth muscle cells with an important role in keeping the atherosclerotic plaque more stable. Oxidized LDLc is toxic for the macrophages, smooth muscle cells and endothelium, with a tremendous role in the initiation and development of atherosclerosis, being also localized within the atherosclerotic lesions [63]. Another study investigated the cytoprotective effects of ascorbic acid by inducing cell apoptosis of human smooth muscle cells and then observing the attenuation effect of pre-administration of vitamin C, providing protection against cytotoxicity of LDLc and lipid hydroperoxides [64].

The recommended intake levels of vitamin C of 75 mg for women and 90 mg for men, per day, may not be enough given the quantity of dietary ascorbic acid needed to have a therapeutic effect in CVD. Most studies used supplementation doses of 500–1000 mg vitamin C per day in order to achieve relevant results [65,66].

To evaluate the oxidative stress on endothelium, another research aimed for the effects of vitamin C on blood pressure in dipper and non-dipper hypertensive patients. Under controlled conditions, vitamin C was administered before the intraarterial infusing with acetylcholine, saline and sodium nitroprusside at increasing doses. This trial showed that ascorbic acid protects the vascular wall from free radical lipid peroxidation and from vasodilatation induced by acetylcholine [67].

Vitamin C acts as an antioxidant and as an enzyme cofactor, with primary biological effects. Oxidized LDLc is highly atherogenic and induces the recruitment of monocyte-macrophages to the endothelial wall, which is a key mechanism in the subclinical atherosclerotic onset. Ascorbic acid effect may start in early stages of vascular dysfunction and, as a result, of atherosclerosis, though vitamin C seems to improve the inflammatory status or blood pressure [68,69].

On the other hand, long time supplementation with vitamin C had no effect on major CV events in specific clinical trials, while even one study remarked a higher all-cause mortality in the antioxidant group but on a population with present CVD [66,70]. The initial hypothesis was sustained by some cohort studies that generally showed an inverse correlation between vitamin C level and population with manifest atherosclerotic CVD or diabetes [66,71]. The lack of data consistency should be regarded from different aspects: apparent different effect of dietary vitamin C from supplementation, rather limited follow-up, reliance on self-reported nutritional questionnaires, as well as the presence of multiple human antioxidant mechanisms that have not been fully understood. The low vitamin status may allow a faster progression of the disease so the expected effects in subjects with normal plasma concentrations of vitamin C seem minimal [72]. However, some data show a direct link between vitamin C and endothelial function as it may reduce the arterial stiffness through antioxidant and anti-inflammatory effects leading to potential stabilization of the atherosclerotic plaque [21,71]. Based on such premises, there are currently few studies that examined subclinical modifications in persons without CVD, but the results for vitamin C look promising and plead for further research (Table 3).

### 2.4. Vitamin B

B vitamins are a complex of eight water-soluble compounds with chemically distinct structure and essential role in the cell metabolism as they act as coenzymes in a variety of anabolic and catabolic enzymatic reactions [76]. They often coexist in the same type of food, but individual B supplements can be used separately, as each one presents specific properties, being either a cofactor for essential metabolic processes or a precursor in the same area [77]. The following paragraphs aim to assess the potential role of each vitamin B subgroup on the atherosclerotic process.

#### 2.4.1. Vitamin B1, or Thiamine (Aneurin)

This is a coenzyme with an active role in processing sugar, fats and amino acids. This micronutrient takes four forms in the human body and is essential in many oxidation-reduction reactions such as the Krebs cycle (for adenosine triphosphate production) or the glucose metabolism. It can be obtained from many types of food, such as cereals, red meat, seeds, nuts, yeast, but high temperature and pH denaturate thiamine, as well as food processing, losing all its beneficial properties [78].

Thiamine loss is closely connected with creatinine clearance since the serum excess that is not bound with protein is eliminated through the distal nephrons. This is the explanation why diuretics are the main cause of thiamine deficiency in patients with CVD. Moreover, the levels of serum thiamine presented a negative correlation with triglycerides and LDLc [79].

In the development of atherosclerotic plaque, an essential role is represented by the proliferation of arterial smooth muscle cells through glucose and insulin mediated mechanisms. Thiamine, as an essential element in glucose metabolism, has a protective role against the atherogenic process, counteracting the effect of high glucose doses that have a tremendous damaging effect over the vascular wall by inducing chronic inflammation through multiple pathways, such as lipid peroxidation, injury or infection that tend to increase the risk of dyslipidemia [80].

An improvement in cardiac and hemodynamic functions as well as a decrease in vascular resistance have been detected after the administration of intravenous thiamine. In patients with hyperglycemia, the endothelium-dependent vasodilatation improved, being recommended the administration of thiamine as routine for slowing down atherogenesis and for the improvement of endothelial functions [81].

A recent trial assessed the endothelium-dependent vasodilatation in three categories of patients: healthy, with impaired glucose tolerance, and with non-insulin-dependent diabetes using duplex ultrasound to measure brachial artery vasoactivity. The study concluded that the effects of thiamine are not based on the glucose-lowering mechanism, given that there was no change under normoglycemia. However, in hyperglycemic patients prone to accelerated atherosclerosis, the effects of vitamin B1 were outstanding by improving the endothelial function and lowering the development or the progression of subclinical atherosclerotic disease [81].

Other studies revealed that supplementation with vitamin B1 has dramatically reduced the vascular inflammation, with negative correlations between high levels of thiamine and LDLc, respectively, triglycerides. Therefore, it may be considered that chronic administration of vitamin B1 can delay the atherosclerotic process [82].

Vitamin B1 can be used both as a marker and as a therapeutic element, with presumed role in the development and also in the prognosis of atherosclerotic vascular pathology. Routine administration could improve vascular function in hyperglycemic persons that represent a target group in many trials assessing subclinical atherosclerosis [83].

#### 2.4.2. Vitamin B2, or Riboflavin

Riboflavin is a water-soluble vitamin, essential for human health, that is closely connected with energy production by acting as a cofactor through its two forms: flavin adenine mononucleotide (FMN) and flavin adenine dinucleotide (FAD). It is found in a wide variety of foods, mostly meat and dairy, most of the time in its cofactor forms, attached to proteins. Deficiency of vitamin B2 is more prevalent in under-developed countries and often occurs along with other nutrient deficiencies [84].

The antioxidant effects of riboflavin have not been widely studied. However, research showed the critical action of vitamin B2 in its FAD coenzyme form in the glutathione redox cycle. An increase of oxidized glutathione can lead to cellular damage and may occur during riboflavin deficiency meaning that an increase in lipid peroxidation can occur, with direct effects in the atherogenic pathways [84].

Many animal studies have shown improvement in subjects with riboflavin deficiency, but in the only human trial that was conducted, there was no significant difference between healthy subjects and riboflavin-deficient subjects [85]. Still, riboflavin status affects the production of lipid peroxides, which means that oxidative stress is closely connected to vitamin B2 [86]. These findings prove that riboflavin has a key role in human body defense system as an antioxidant and the low intake can affect the atherosclerotic process by misbalancing the oxidant-antioxidant ratio [87].

#### 2.4.3. Vitamin B3, or Niacin or Nicotinic Acid (NA)

Niacin is a water-soluble vitamin with essential role in cellular energy and metabolism. It is found in a large variety of foods such as meat, grains and dairy. Niacin deficiency is rare in developed countries and can lead to pellagra, a human disease characterized by symptoms such as high sensitivity to sunlight, dermatitis, diarrhea, depression and dementia [88].

NA has an essential role in metabolism as it is the precursor of nicotinamide adenosine dinucleotide (NAD+) and nicotinamide adenine dinucleotide phosphate (NADP+) that have key roles in glycolysis, tricarboxylic acid cycle, the respiratory chain and other metabolic processes that require electron transport. They also have a major impact in cellular processes such as DNA repair and genomic structure [88].

Niacin deficiency is involved directly in the oxidative stress by altering the redox balance of the cell, explaining thus the decreased levels of antioxidant enzymes and by interfering in the regeneration of glutathione, with a direct effect on deactivating peroxides as a response to stress [89].

The effect on subclinical atherosclerosis has been intensively studied since the revealing of the niacin ability to increase plasma HDLc and to decrease the levels of proatherogenic lipids and lipoproteins, thus intervening in the atherogenic process. HDLc is an independent risk factor in CVD, with predictive capacity in the atherosclerotic vascular disease, with lower levels found especially in healthy women and less in men [90]. NA also prevents endothelial dysfunction by improving the response to proinflammatory and prothrombotic factors of the vascular wall and by its effect over the substances released from the endothelial cells that inhibit vasorelaxation [91]. Niacin hypolipidic effect also reduces blood thickness and antiplatelet aggregation, explaining the direct effect on subclinical atherosclerosis [92].

Many studies have focused on niacin effect on atherosclerotic plaque, most of them using associations with statins or other pharmaco-active substances in CVD area, all of them with promising results [93]. However, only one trial used niacin alone versus placebo. The study was conducted over 52 weeks and on 50 patients with metabolic syndrome but no CVD, randomized to either placebo or 1 g extended-release niacin. After this time period, by cIMT assessment, the endothelial function increased by 22% in the niacin receiving group, while no change was seen in the placebo group. Thus, therapy with extended-release niacin may improve the metabolic parameters, by increasing HDLc and reducing triglycerides, with effects on atherosclerotic plaque reduction [94].

#### 2.4.4. Vitamin B5, or Pantothenic Acid (PA)

Pantothenic acid is a precursor for coenzyme A (CoA), essential for biochemical reactions in human body such as Krebs cycle, fatty acid metabolism or β-oxidation [95]. The role of PA in atherogenesis is not fully elucidated, but it may have a key effect on reduction of oxidative stress by increasing glutathione synthesis [96].

C reactive protein (CRP), a circulating inflammatory marker, has a controversial role in the atherosclerotic disease, but it can transform oxidized LDLc into foam cells and can be a stable biomarker for low-grade inflammation, especially found in subclinical atherosclerosis. Several studies have shown a cross-link between the levels of PA and CRP as well as inverse correlation with LDLc and triglycerides levels, but the mechanisms are still unknown [97,98].

Pantethine is physiologically synthesized in the human body from PA and is safe and effective in low and moderate CVD risk, being used as nutritional supplement since 1992, as it showed a significant lowering of LDLc and total cholesterol serum concentrations [99]. Data revealed that a 16-week treatment with pantethine reduced LDLc and total cholesterol as compared to placebo, with a significant decrease of the lipid profile in women. Women also had a better decrease of non-HDLc level compared to men who had better results on LDLc fraction [100].

High concentrations of plasma lipids play an essential role in atherogenesis and progression of atherosclerosis. Vitamin B5, as a precursor of CoA, favorably affects triglycerides synthesis and lipoprotein metabolism, contributing to the onset and development of subclinical atherosclerosis.

#### 2.4.5. Vitamin B6

This is a water-soluble vitamin that can be found in various foods such as meat, whole grains, vegetables, fruits and nuts. It has six isoforms, pyridoxal 5′-phosphate (PLP) being the most active. PLP acts as coenzyme for various reactions such as amino acid and carbohydrate metabolisms. Vitamin B6 may have proved a preventive role in CVD, stroke and thrombosis [101,102,103].

Still, trials must prove that low serum vitamin B6 is an independent risk factor for CVD. Low serum PLP appeared to be connected with the imbalance of lipid metabolism. With a direct role in the metabolism of homocysteine, which is involved in atherothrombotic vascular disease by causing direct endothelial damage, increasing oxidative stress, negatively altering the endothelial function and thrombogenesis, PLP might have a multifactorial influence on CVD [104].

Furthermore, PLP is also associated with circulating CRP and other oxidative stress markers that are known to reduce superoxide radical effects in endothelial cells. Also, low vitamin B6 concentrations decrease the glutathione, alter the antioxidant defense system and lead to inflammation, a crucial mechanism in subclinical atherosclerosis [105,106].

#### 2.4.6. Vitamin B7, or Biotin

Biotin is a vitamin found naturally in meat, eggs, cereals and vegetables. It is a cofactor for a variety of carboxylation reactions and is involved in fatty acids metabolism and gluconeogenesis [107]. Biotin deficiency is extremely rare, and even though it might influence the prevention of atherogenesis or atherosclerosis, there are no current studies to prove that yet [108].

#### 2.4.7. Vitamin B9, or Folic Acid

Folic acid presents a beneficial role after acute or chronic administration in various CV predictive markers such as blood pressure, arterial stiffness, endothelial function and pro-thrombotic activity by lowering homocysteine levels and influencing the endothelial nitric oxide synthase (eNOS) [109].

Folic acid also has independent effects on atherogenesis. Chronic administration enhanced the endothelial function, but the homocysteine concentration did not change, these effects being mediated by folic acid and eNOS [110].

Research data support the premise that vitamin B9 may be an effective therapeutic option to reduce the inflammatory response in subclinical atherosclerosis by decreasing the plasma concentration of homocysteine or by the direct interaction with eNOS to reduce production of reactive oxygen species.

Trials on cigarette smokers, otherwise healthy patients, showed that supplementation with folic acid on a 4-week trial reduced significantly the concentration of homocysteine and fibrinogen as compared to placebo [111].

Another trial used 1-year folic acid supplementation in order to explain the correlation between vitamin B9, hyperhomocysteinemia and the proinflammatory status in high CVD risk. 530 men and postmenopausal women with hyperhomocysteinemia at the beginning of the study have been randomized. The effect on inflammatory markers was not significant, revealing that it might be another mechanism for vitamin B9 protective effect [109,112].

#### 2.4.8. Vitamin B12, or Cobalamin

Cobalamin is a water-soluble vitamin used in cell metabolism and is naturally acquired through diet, especially from animal products, being essential for normal functioning of all human tissues. For a good absorption, the stomach, pancreas and ileum must be physiologically intact, otherwise vitamin B12 deficiency may occur [113].

Homocysteine and methylmalonic acid are metabolites of cobalamin, with key roles in atherogenic processes. Low levels of serum vitamin B12 have direct effects on subclinical atherosclerosis by mediating reactions such as the extraction of energy from fat and protein in the mitochondria or the conversion of homocysteine to methionine. These mechanisms reveal how multiple organ-systems can be affected by vitamin B12 deficiency. The clinical manifestation is linked to the severity of organ-system damage but is usually asymptomatic [114].

Hyperhomocysteinemia associated to nutritional deficiency is mild but linked with a high risk of atherogenesis induced by endoplasmic reticulum stress, proinflammatory response and oxidative stress. A study on 158 healthy siblings of patients with premature atherothrombotic disease revealed that lowering the homocysteinemia by cobalamin administration showed a decreased risk of atherosclerotic events as compared to placebo group [115].

By comparing genders, high concentrations of homocysteine were found in males that possessed a higher risk of atherothrombotic process development. However, lowering serum homocysteine concentration was equal in both males and females, with good appliance for future therapeutical applications of vitamin B12 [116].

Even though signs and symptoms of vitamin B12 deficiency are not specific enough for a fast diagnosis, prompt vitamin administration is crucial before irreversible lesions install. Accurate diagnosis remains controversial and the screening is not reliable. Nonetheless, since the treatment is easy and safe, vitamin supplementation should be established at least in persons with risk factors of developing atherothrombotic vascular disease [113,117].

Overall, to the best of our present knowledge, there is no study that has established a specific B vitamin supplementation effect on subclinical atherosclerosis evolution in patients with no CVD at baseline. However, in most clinical trials, there was given a “cocktail” of vitamin B subtypes, most of them including B6, B9, B12. Most interventional studies regarded patients with advanced renal disease as folic acid and B12 may have beneficial effects on renal disease.

The meta-analysis results showed that vitamin B supplementation had no effect on lowering CVD events though, in some subgroup of patients, a higher intake of vitamin B seemed to offer a benefit in primary CVD prevention but not in secondary prevention [118]. The inconclusive results of observational and interventional trials with vitamin B might be explained though the different types and doses of supplements, the non-standardized patient evaluation, multiple CVD comorbidities and divergent types of population. However, hopeful data came from the “B-Vitamin Atherosclerosis Interventional Trial” (BVAIT) that aimed to demonstrate whether homocysteine is a marker or the cause of atherosclerotic vascular pathology given that high levels of this amino acid are associated with high CVD risk. By measuring cIMT and the aortic and coronary artery calcium score, high doses of vitamin B supplementation (composed of folic acid 5 mg + vitamin B12 0.4 mg + vitamin B6 50 mg) reduced the progression of subclinical atherosclerosis in patients with no CVD [119].

### 2.5. Vitamin A

Vitamin A (all-*trans*-retinol) is a provitamin found in vegetables that exists in different metabolic states: retinal (retinaldehyde), oxidized forms and conjugations of retinol and retinoic acid. The term retinoid was introduced in 1970 and gathers under its name all chemical compounds that structurally resemble all-*trans*-retinol, with all its metabolites, both natural and synthetic [120,121].

For long time, vitamin A actions used to be focused on proliferative disorders, mainly around cancer and dermatologic diseases. However, new findings have proved that vitamin A possesses certain influence on the CV system and metabolic pathologies, being involved in angiogenesis, oxidative balance and cellular growth [122].

Vitamin A is not a water-soluble micronutrient, being itself a lipid. This explains why it is mainly found bound to specific vitamin A-binding proteins or within the cells in lipid droplets, proteins that are highly involved in the pathogenesis of obesity and diabetes [122]. The processing of vitamin A in the intestine after dietary intake is similar with triglycerides and cholesterol, but once absorbed, the metabolism, as well as its storage, is unique. Vitamin A is essential for mediating various physiological processes in the human body, some of them related to cholesterol and triglycerides metabolism, contributing to pathogenesis of metabolic diseases when dysregulated [123].

There is a connection between retinoids, the arterial responses to injury and atherogenesis. Animal studies proved an impressive decrease of atheromatous plaque after four days of vitamin A administration as well as a reduction of the neointimal injury by modulating smooth muscle cell migration and proliferation with essential role in atherogenesis [124]. Retinoids limit the vascular smooth muscle cell proliferation and regulate apoptosis, while lesions appear when there is an imbalance between smooth muscle cell proliferation apoptosis and cell death [125]. Retinoids also have a direct role in the coagulation system by promoting fibrinolysis and decreasing the pro-thrombotic effects of the vascular endothelium [126]. Furthermore, vitamin A regulates favorably the inflammatory mechanisms by decreasing endothelial adhesion molecule expression with a direct role in all stages of atherogenesis [127].

In observational and interventional cohort studies, vitamin A supplementation was not associated with incremental effect on CVD events [128]. Nonetheless, there is data that suggest that the protective effects of vegetables and fruits against CVD may be due to increase lutein consumption. It presents antioxidant properties and prevents the activation of plasma damaging complement factors. This was speculated and assessed by comparing two different populations, Mediterranean and Northern ones, which showed that the healthier cardiometabolic features displayed by the first population may be due to the higher concentration of plasma lutein as other antioxidants did not differ significantly [129].

Besides lutein and carotenoids, retinol-binding protein 4 (RBP4) might present beneficial effect when it is additionally supplemented. By measuring the circulating retinol-binding protein 4 (RBP4), transthyretin (TTR) and the cIMT, a link between vitamin A and subclinical atherosclerosis was shown. Further studies have proved that RBP4 links to insulin resistance, diabetes and atherosclerosis, retinol being inversely corelated with cIMT. Higher level of RBP4 was correlated with a specific role in atherogenesis, dietary vitamin A showing a strong protective action on subclinical atherosclerosis [130].

A study on clinically healthy European elder people hypothesized that vitamin A could be linked with atherosclerosis, endothelial function and left ventricular function, based on the new findings that RBP4 concentration correlates with subclinical inflammation in the early stages of infant obesity. The study assessed cIMT and the plaque echogenity, revealing an inverse association between circulating RBP4 concentrations and cIMT which suggests the involvement of RBP4 in the atherogenic mechanism [131].

On the endothelial function, retinoids play an important role over nitric oxide, a potent vasodilator that controls vascular functions, but studies with human component are yet to take place.

A study on children aged 14–18 years showed elevated serum RBP4 concentration in obese patients and strongly association with subclinical inflammation. Changes in lifestyle, such as physical activity and healthy diet, have decreased serum RBP4 concentration and the inflammatory markers, with important clinical implications [132].

The up-mentioned results are summarized in Table 4. Retinoid actions on atherosclerosis involve various cells and complex mechanisms, with multiple effects on the metabolic processes and responses that interact with atherogenesis and, implicitly, with subclinical atherosclerosis. The effects over cells and pathological situations reveal their specific biology, regulating both atherosclerotic and vascular injuries.

### 2.6. Vitamin Supplementation Summary

This review aimed to assess the association between the vitamin intake and subclinical atherosclerosis, trying to determine correlations between beneficial effects of rich-vitamin diets and CVD development. Table 5 aims to offer a summary for the nowadays evidence and the possible recommendations for the vitamin supplementation effect on subclinical atherosclerosis in patients without manifest CVD as well as the major implications in atherogenesis for each vitamin. However, it is worth mentioning that the current CVD prevention guidelines do not recommend specific vitamin supplementation. Nonetheless, being safe and effective to use, with low to none adverse effects, these substances can be extremely valuable in the modern medicine focused on new alternative methods for the prevention of atherothrombotic vascular disease.

Though the vitamin supplementation interventional trials presented divergent results, the lack in heterogeneity of study population, time of monitorization and different selected outcomes may be key arguments for the up-to-date results. As well, there were more studies that tried to focus on increase intake in secondary prevention than in primary prevention. Nonetheless, the interventional trials that regard subclinical atherosclerosis changes in patients without manifest CVD are rather few, but the results are promising and deserve further evaluation as subclinical disease often evolves asymptomatic. Thus, for a better understanding and clear results of vitamin supplementation, future clinical interventional trials should focus more on primary prevention in specific chosen group population. An individual comprehensive evaluation should be carefully assessed at baseline initiation for having similar group characteristics, with the exclusion of several diseases that could alter the vitamin metabolism as renal or hepatic impairment. The follow-up should be conducted on longer periods of time, at least 2–3 years, as the effects seem to be cumulative and time dependent. Further on, the dosage and type of administration should be standardized, and the final study analysis should address, as well, to different subgroups that may additionally benefit from the vitamin intake.

## 3. Conclusions

Although certain clinical trials have shown promising results, there are so far no conclusive data regarding the prevention and progression of atherosclerosis based on vitamin supplementation in patients without CVD. However, given their enormous potential in this research area, future trials will certainly bring additional information for a tailored CVD prevention focusing on early stages as subclinical atherosclerosis.

## Figures and Tables

**Table 1:** Effects of vitamin E supplementation on subclinical atherosclerosis in patients without manifest CVD.

Trial	Year	Follow-up	Dose	Population	Age	Results
Rasool et al. [24]	2008	2 months	112, 224, 448 IU vitamin E, daily	36 healthy subjects	23.9 ± 0.39 years	Improvement of pulse wave velocity (PWV) at doses of 224 and 448 IU/day
Hodis et al. (VEAPS) [25]	2002	3 years	400 IU vitamin E, daily	353 healthy subjects with high LDLc	56.2 years (range 40–82 years)	No effect on cIMT progression
Skyrme-Jones et al. [26]	2000	3 months	1000 IU vitamin E, daily	41 type 1 diabetic subjects	23 ± 6 years	Improvement of endothelial vasodilatation function
Salonen et al. (ASAP) [18]	2000	3 years	272 IU vitamin E, daily	115 healthy subjects	range 45–69 years	Improvement of cIMT

**Table 2:** Effects of vitamin D supplementation on subclinical atherosclerosis in patients without manifest CVD.

Trial	Year	Follow-up	Dose	Population	Age	Results
Borgi et al. [39]	2017	8 weeks	50.000 IU D2	84 overweight/obese adults	37 ± 12.3 years	No improvement on endothelial function
Kumar et al. [40]	2017	16 weeks	2 doses of 300.000 IU D3	117 subjects with chronic kidney disease	43.17 ± 11.79 years	Improvement on FMD and PWV
Raed et al. [41]	2017	16 weeks	600, 2000, 4000 IU/day D3	70 overweight patients	26.2 ± 9.8, 24.4 ± 8.7, 25.5 ± 9.0 years	Improvement on PWV
Bressendorff et al. [42]	2016	16 weeks	3000 IU D3	40 healthy subjects	41.0 ± 9.05 years	No effect on PWV
Farouhi et al. [43]	2016	16 weeks	100.000 IU D2 / 100.000 IU D3	160 type 2 diabetic subjects	53.5 ± 8.7 years (D2); 52.5 ± 8.2 years (D3)	Improvement on PWV
Zaleski et al. [44]	2015	6 months	400 IU/day vs. 4000 IU/day D3	40 prehypertensive subjects	34.8 ± 12.8 years (low dose); 40.4 ± 7.5 years (high dose)	No effect on PWV
Martins et al. [45]	2014	12 weeks	100.000 IU D3	130 overweight/obese subjects	range 18–70 years	No effect on augmentation index
Breslavsky et al. [46]	2013	1 year	1000 IU D3	32 type 2 diabetic subjects	69 ± 9 years	Improved augmentation index
You et al. [47]	2013	3 months	5000 IU D3	12 type 2 diabetic subjects	65 ± 8 years	No effect on FMD
Harris et al. [48]	2011	4 months	60.000 IU D3	45 overweight patients	29 ± 2 years	Improved FMD
Sugden et al. [49]	2008	8 weeks	100.000 IU single dose D2	34 type 2 diabetic patients	64.9 ± 10.3 years	Improved FMD

**Table 3:** Effects of vitamin C supplementation on asymptomatic CVD subjects and subclinical atherosclerosis.

Trial	Year	Follow-up	Dose	Population	Age	Results
Hildreth et al. [73]	2014	6 months	0.06 g/kg of fat free mass–max 7.5 g vitamin C, intravenous single dose	97 healthy women	22–70 years	Improvement of arterial stiffness (measured by carotid artery compliance)
Mullan et al. [74]	2002	4 weeks	500 mg vitamin C/day, orally	30 type 2 diabetic subjects	61.0 ± 6.5 years	Improvement of arterial stiffness (augmentation index; time to wave reflection)
Wilkinson et al. [75]	2001	6 h	2000 mg vitamin C, orally	8 healthy subjects	29 (20–42) years	Improvement of augmentation index, but not on PWV

**Table 4:** Effects of vitamin A supplementation on subclinical atherosclerosis in patients without manifest CVD.

Trial	Year	Population	Age	Results
Huang et al. [133]	2012	709 postmenopausal women	52.9 ± 2.6 years	No improvement on cIMT
Inglesson et al. [131]	2008	1008 healthy subjects	70 years	Improvement on arterial stiffness (cIMT)

**Table 5:** Summary table for vitamin supplementation effect on subclinical atherosclerosis in subjects without CVD.

Vitamin Supplementation	Level of Evidence	Recommendation for Subclinical Atherosclerosis Improvement	Main Implications in Atherogenesis	
E	+++	+++	↓ LDLc ↓ oxidative stress ↓ cellular aggregability	↓ smooth muscle cell proliferation ↑atheroma plaque stabilization
D	+++++	++	↓ inflammation ↓ oxidative stress ↓ smooth muscle cell proliferation	↓ renin-angiotensin-aldosterone system activity
C	+++	+++	↓ oxidized LDLc ↓ oxidative stress ↓ inflammation	↓ monocyte endothelial adhesion ↑ nitric oxide ↑ antioxidant effect
B complex	+	+	↓ homocysteine ↓ LDLc ↓ inflammation	↓ cellular aggregability ↑ endothelial-dependent vasodilatation ↑ nitric oxide
A	+	+	↓ oxidized LDLc ↓ inflammation	↓ smooth muscle cell proliferation ↑ nitric oxide

+ means the importance—where + is the least indicated/studied and +++++ is the most indicated/studied; ↓ means diminish; ↑ means augments.
